# Antimicrobial resistance profiles of *Escherichia coli* from swine farms using different antimicrobials and management systems

**DOI:** 10.14202/vetworld.2021.689-695

**Published:** 2021-03-19

**Authors:** Pramualchai Ketkhao, Sukanya Thongratsakul, Pariwat Poolperm, Chaithep Poolkhet, Patamabhorn Amavisit

**Affiliations:** 1 Center for Agricultural Biotechnology, Kasetsart University Kamphaeng Saen Campus, Nakhon Pathom, Thailand; 2 Center of Excellence on Agricultural Biotechnology: (AG-BIO/PERDO-CHE), Bangkok, Thailand; 3 Department of Veterinary Public Health, Faculty of Veterinary Medicine, Kasetsart University, Nakhon Pathom, Thailand; 4 Faculty of Veterinary Medicine, Kasetsart University, Kamphaeng Saen Campus, Nakhon Pathom, Thailand; 5 Department of Microbiology and Immunology, Faculty of Veterinary Medicine, Kasetsart University, Bangkok, Thailand

**Keywords:** antimicrobial resistance, colistin, swine farms, *mcr*

## Abstract

**Background and Aim:**

The emerging of antimicrobial-resistant foodborne bacteria is a serious public health concern worldwide. This study was conducted to determine the association between farm management systems and antimicrobial resistance profiles of *Escherichia coli* isolated from conventional swine farms and natural farms. *E. coli* isolates were evaluated for the minimum inhibitory concentration (MIC) of 17 antimicrobials, extended-spectrum beta-lactamase (ESBL)-producing enzymes, and plasmid-mediated colistin-resistant genes.

**Materials and Methods:**

Fecal swabs were longitudinally collected from healthy pigs at three stages comprising nursery pigs, fattening pigs, and finishers, in addition to their environments. High-generation antimicrobials, including carbapenem, were selected for the MIC test. DNA samples of colistin-resistant isolates were amplified for *mcr-1* and *mcr-2* genes. Farm management and antimicrobial applications were evaluated using questionnaires.

**Results:**

The detection rate of ESBL-producing *E. coli* was 17%. The highest resistance rates were observed with trimethoprim/sulfamethoxazole (53.9%) and colistin (48.5%). All isolates were susceptible to carbapenem. Two large intensive farms that used colistin-supplemented feed showed the highest colistin resistance rates of 84.6% and 58.1%. Another intensive farm that did not use colistin showed a low colistin resistance rate of 14.3%. In contrast, a small natural farm that was free from antimicrobials showed a relatively high resistance rate of 41.8%. The majority of colistin-resistant isolates had MIC values of 8 mg/mL (49%) and ≥16 mg/mL (48%). The genes *mcr-1* and *mcr-2* were detected at rates of 64% and 38%, respectively, among the colistin-resistant *E. coli*.

**Conclusion:**

Commensal *E. coli* were relatively sensitive to the antimicrobials used for treating critical human infections. Colistin use was the primary driver for the occurrence of colistin resistance in swine farms having similar conventional management systems. In the natural farm, cross-contamination could just occur through the environment if farm biosecurity is not set up carefully, thus indicating the significance of farm biosecurity risk even in an antimicrobial-free farm.

## Introduction

The presence of bacteria with antimicrobial resistance (AMR) in food-producing animals is believed to be an important cause of AMR dissemination in humans and the environment. Antimicrobial agents are commonly used in livestock for several purposes, including therapeutic, metaphylaxis, and prophylaxis [1]. Following a global action plan to tackle AMR, a national action plan has been introduced in Thailand. The use of all antimicrobial agents as growth promoters in swine farms has been prohibited since 2015 [2], and in 2018, colistin use was restricted, making it applicable for veterinary prescription alone [3].

In Thailand, swine farms have different management systems and production sizes, ranging from large farms with fully vertically integrated systems to small backyard farms. The scale of the farming systems was categorized according to the number of animals per farm [4]. A farm raising <50 animals was considered as a small-scale farm, and with 500-5000 swine was considered as a medium-sized farm. A large-scale farming system had more than 5000 animals. In general, large intensive farms preferred standard operating procedures and facilities to minimize animal infections. Examples of antimicrobial agents used in small and medium Thai swine farms include penicillin, streptomycin, amoxicillin, enrofloxacin, oxytetracycline, kitasamycin, and colistin [5]. Before prohibition, colistin sulfate was commonly used for decades in swine farms to treat gastrointestinal tract infections caused by *Escherichia coli* and *Salmonella* [6]. Colistin is a member of the polymyxin group that has been used to treat Gram-negative bacterial infections. Excessive global use of colistin in human and veterinary medicine has resulted in increased colistin resistance [7]. A report on plasmid-mediated colistin resistance, controlled by the *mcr-1* gene that could be horizontally transferred, raised the awareness about bacteria with AMR [8]. Several *mcr* variants, including *mcr-1*, *mcr-2*, *mcr-3*, *mcr-4*, *mcr-5*, *mcr-6*, *mcr-7*, *mcr-8*, *mcr-9*, and *mcr-10*, have been reported [8-17].

This study was conducted to investigate the association between farm management systems and AMR profiles of *E. col*i collected longitudinally from four swine farms. The distribution of *mcr-*1 and *mcr-*2 genes and their relationship with colistin minimum inhibitory concentration (MIC) was also evaluated.

## Materials and Methods

### Ethical approval and Informed consent.

The animal ethics were approved by Animal Care and Use for Scientific Research Kasetsart University (ACKU59-VET-002). Informed consent was obtained from the farm owner before the study.

### Sample collection, farm information and study period

Samples from four swine farms were longitudinally collected at three stages of production, that is, nursery pigs (1 month old), fattening pigs (3 months old), and finishers (5 months old). Color marks or ear tags were used to individually identify the selected piglets. Each stage of the animal and the environment samples was collected concurrently. The study period per individual production cycle lasted approximately 6 months, from nursery pigs to finishers. The sampling periods for farms A, B, C, and D were June-November 2016, July-December 2016, August 2016-January 2017, and January-June 2017, respectively. Of the 347 samples collected from farm A (n=85), farm B (n=87), farm C (n=87), and farm D (n=88), 107 samples were fecal swabs from farm A (n=25), farm B (n=27), farm C (n=27), and farm D (n=28), and 60 samples each were collected from boot swabs, floor swabs, clean water, and wastewater.

The farms were located in the central part of Thailand, which had the highest density of swine industry. Farms A, B, and C had more than 5000 fattening pigs per farm and were considered as large-scale intensive farms with conventional farm management. In contrast, farm D was a small-scale, natural farm that had <50 fattening pigs. The farm owners or managers were interviewed for information on general biosecurity management and antimicrobial applications, especially colistin (Table-1).

**Table-1:** Farm biosecurity and antimicrobial usage of four swine farms.

Determinant	Farm A	Farm B	Farm C	Farm D
Farm biosecurity				
Fencing	Yes	Yes	Yes	Yes
Boot separation	Yes	Yes	Yes	Yes
Access to the farm	Restricted	Restricted	Restricted	Freely
Pest control	Yes	Yes	Yes	No
Separated workers for each swine housing	Yes	Yes	Yes	No, they worked in every housing
Boot cleaning before and after entering housing	Yes, they were cleaned with disinfectant			Yes, they are cleaned with water
Outside vehicle cleaning before entering to the farm	Yes, the vehicle was cleaned with disinfectant			No cleaning
Manure system	Covered lagoon	No	Covered lagoon	No, they replaced bedding every 3 months
Cloth changing	Wearing white coat	No	Yes	No
Antimicrobial use				
Feed medicated for nursery swine	Amoxicillin 300 ppm	No	Amoxicillin 300 ppm	No
	Colistin 150 ppm		Chlortetracycline 400 ppm	
	Tylosin 200 ppm		Colistin 150 ppm	
	Zinc oxide 3000 ppm		Tiamulin 200 ppm	
Therapeutic treatment (injection)	Amoxicillin	Gentamicin	Amoxicillin	No
	Ampicillin and colistin		Ceftriaxone	
	Gentamicin		Enrofloxacin Gentamicin	

### Isolation and antimicrobial susceptibility test of *E. coli*

The fecal swabs, boot swabs, and floor swabs were directly cultured on chromogenic coliform agar (Merck Millipore, Germany), and wastewater and clean water were cultured for *E. coli* according to the International Organization for Standardization (ISO) 9308-1[18]. One colony from each type of positive specimen was selected for further analysis. A total of 206 *E. coli* selected from fecal swabs (n=48), boot swabs (n=44), floor swabs (n=47), wastewater (n=44), and clean water (n=23) were analyzed to evaluate the MIC of the following 17 antimicrobials using the AST-N194 test kit in the VITEK2 compact automated machine (bioMérieux, Marcy-I’Etoile, France): Amoxicillin/clavulanic acid, ceftazidime, cefoperazone/sulbactam, cefpirome, cefotaxime, cefoxitin, doripenem, imipenem, meropenem, amikacin, gentamicin, netilmicin, ciprofloxacin, moxifloxacin, tigecycline, colistin, and trimethoprim/sulfamethoxazole. Extended-spectrum beta-lactamase (ESBL)-producing *E. coli* were also screened using the kit. *E. coli* ATCC 25922 and *Klebsiella pneumoniae* ATCC 700603 were used as the quality control strains. AMR breakpoints were interpreted according to the criteria of the Clinical and Laboratory Standards Institute [19], except the moxifloxacin breakpoint that was interpreted according to the VITEK 2 ­system software version 7.01 (bioMérieux, Marcy-I’Etoile, France). The colistin breakpoint was interpreted according to the European Committee on Antimicrobial Susceptibility Testing version 4 [20]. The criteria of multidrug-resistant (MDR) isolates described by Magiorakos *et al*. [21] were used to evaluate acquired non-susceptibility to at least one agent in three or more antimicrobial categories.

### Detection of plasmid-mediated colistin-resistant genes

Of the 206 *E. coli*, 100 isolates were resistant to colistin and were tested for *mcr-1* and *mcr-2* genes by PCR. Each PCR assay was performed using 5X Phusion HF buffer (7.5 mM MgCl_2_), 200 μM of each dNTP, 0.5 μM of each forward and reverse primer, 0.02 units of Phusion Hot Start II High-Fidelity DNA polymerase (Thermo Scientific, USA), and 1 mL of DNA template. The *mcr-1* and *mcr-2* genes were amplified using the primers CLR-F (5´-CGGTCAGTCCGTTTGTTC-3´), CLR-R (5´-CTTGGTCGGTCTGTAGGG-3´), MCR2-IF (5´-TGTTGCTTGTGCCGATTGGA-3´), and MCR2-IR (5´-AGATGGTATTTGGTTGCTG-3´) according to the methods previously described by Liu *et al*. [8] and Xavier *et al*. [9], respectively.

### Statistical analysis

Descriptive statistical analysis was conducted to describe the AMR rates, the frequency of *mcr-1* and *mcr-2* detection, and the fundamental information of each farm. Pearson’s Chi-square test was used to analyze the AMR rates and the colistin-resistant genes between the farms using the NCSS 11 software (NCSS, Kaysville, UT), and p<0.05 was considered to be statistically significant.

## Results

The three large-scale intensive farms (A, B, and C) were conventional farms with standard biosecurity programs, including restricted access, worker separation, pest control, fencing, and outside vehicle cleaning (Table-1). In farms A and C, colistin was supplemented in nursery feeds at a concentration of 150 ppm to control post-weaning diarrhea caused by *E. coli*. Amoxicillin (300 ppm), tylosin (200 ppm), and zinc oxide (3000 ppm) were supplemented in feeds in farm A. In comparison, amoxicillin (300 ppm), tiamulin (200 ppm), and chlortetracycline (400 ppm) were used in farm C. However, farm B did not use colistin or other antimicrobial agents in animal feeds, but other injectable antimicrobials, including gentamicin, were used for treatment. Remarkably, farm D with a small-scale nature was free from antimicrobial agents as the sick animals were treated using herbal plants.

The MIC ranges of the 17 antimicrobials and the resistance rates are presented in Table-2. The majority of *E. coli* isolates (81.6%; 168/206) were resistant to at least one antimicrobial agent, whereas 18.4% (38/206) were sensitive to all the tested antimicrobial agents. Among the 206 tested isolates, high rates of resistance to trimethoprim/sulfamethoxazole (53.9%) and colistin (48.5%) were found. The resistance to gentamicin (30.6%), cefotaxime (19.4%), ­moxifloxacin (17.5%), and ciprofloxacin (13.1%) was moderate. Low rates of resistance were found for ceftazidime (5.3%), cefoxitin (5.3%), amoxicillin/clavulanic acid (2.9%), netilmicin (1.9%), tigecycline (1%), and cefpirome (0.5%). All isolates were susceptible to carbapenem agents (doripenem, imipenem, and meropenem), cefoperazone/sulbactam, and amikacin. There were 35 ESBL-producing isolates (17%), and all were resistant to cefotaxime. Moreover, 15 and 11 ESBL-positive isolates were found in farms A and C, respectively, whereas farms B and D had only 3 and 6 ESBL-positive isolates, respectively.

**Table-2:** The MIC distribution of 17 antimicrobial agents among 206 *E. coli* isolated from swine fecal swabs and environmental samples from four swine farms.

Agents^a^	Number of isolates													Resistances
	
	MIC (µg/mL)													(%)

	0.12	0.25	0.5	1	2	4	8	16	20	32	64	320	Breakpoints	
AMC					6	144	30	20		6			≥32	2.9
CAZ				177		16	2	10			1		≥16	5.3
CFP							192	13		1		≥64	0
CPO				171		16	4	9		5	1		≥64	0.5
CTX				165	1	6	6	6		1	21		≥4	19.4
CX						160	23	12		1	10		≥32	5.3
DOR	205	1											≥4	0
IMP		205			1								≥4	0
MEM		206											≥4	0
AK					182	24							≥64	0
GN				139	1		3	63					≥16	30.6
NET				117	27	10	39	9		4			≥32	1.9
CIP		131	22	21	5	27							≥4	13.1
MXF^b^		86	12	5	62	5	36						≥8	17.5
TGC			171	6	12	15	2						≥8	1.0
CL^c^			103		3	3	49	48					≥4	48.5
SXT									95			111	≥64	53.9

a AMC=Amoxicillin/clavulanic acid, CAZ=Ceftazidime, CFP=Cefoperazone/sulbactam, CPO=Cefpirome, CTX=Cefotaxime, CX=Cefoxitin, DOR=Doripenem, IMP=Imipenem, MEM=Meropenem, AK=Amikacin, GN=Gentamicin, NET=Netilmicin, CIP=Ciprofloxacin, MXF=Moxifloxacin, TGC=Tigecycline, CL=Colistin and SXT=Trimethoprim/sulfamethoxazole. Vertical bold lines are antimicrobial breakpoints followed CLSI (2014),

b VITEK 2 automated machine (bioMérieux, Marcy-I’Etoile, France) and

c EUCAST version 4.0. Gray fields represent level of concentration referring resistance. MICs equal to or lower than lowest concentration and MIC equal to or higher than tested are single and double underlined, respectively

Regarding the AMR patterns, 35.7% of the isolates exhibited resistance to single drugs, including SXT, CL, GN, or CTX. MDR isolates were detected at a rate of 26.2% (54/206) in this study. The MDR R-type CTX-GN-CL-SXT was found primarily at 24.1% (13/54) followed by CTX-GN-CL at 7.41% (4/54) and CTX-GN-CIP-MXF-CLSXT at 7.41% (4/54). However, the other MDR R-types were found in only 1-3 isolates in each pattern. In terms of each farm, MDR isolates were frequently found in farm C at 48.1% (25/52), followed by farms A (39.5%; 17/43), D (18.2%; 10/55), and B (3.6%; 2/56).

Regarding colistin-resistant isolates, farm C showed the highest proportion of 84.6% (44/52), followed by farms A (58.1%; 25/43), D (41.8%; 23/55), and B (14.3%; 8/56). The overall resistance rate was not related to the sample source (p>0.05). The resistance rates were 56.3% (27/48) in fecal swabs, 40.9% (18/44) in boot swabs, 51.1% (24/47) in floor swabs, 45.5% (20/44) in wastewater, and 47.8% (11/23) in clean water.

As the samples were collected 3 times longitudinally, the transition rate of colistin resistance was examined through the three production stages. Colistin resistance in farm A was the highest among nursery pigs (94.4%), decreased in fattening pigs (63.6%), and dramatically reduced among finishers (7.1%). The resistance rates in farm D increased from the nursery stage (44.4%) to the fattening stage (72.2%) and then decreased in finishers (10.5%). In farm C, the isolates exhibited high rates of colistin resistance in every stage (100%, 83.3%, and 72.2%, respectively), whereas the isolates collected from farm B exhibited low rates of resistance in all stages (0%, 33.3%, and 10.5%, respectively). In the four farms, the resistance rates decreased in each farm when the samples were collected from finishers.

Of the total 100 colistin-resistant isolates, 49 and 48 isolates had MIC values of 8 and ≥16 mg/mL, respectively (Table-2). DNA amplification of the 100 colistin-resistant *E. coli* revealed that 64 isolates harbored the *mcr-1* gene and 38 isolates harbored the *mcr-2* gene (Table-3). In total, 26 isolates carried both genes, whereas 24 isolates were negative to both genes. The presence of *mcr-1* and *mcr-2* genes was not related to colistin MIC (p>0.05).

**Table-3:** Comparison of resistance rates and colistin MIC of 206 *E. coli* isolated from four swine farms, *mcr-1* and *mcr-2* genes were amplified in 100 colistin-resistant isolates.

Farm	Resistance rates			*mcr* carrying CL resistant isolates	Colistin MIC (μg/mL)					
	
	Number of isolates (%)				Number of isolates					Total
	
	ESBL^a^	SXT^b^	CL^c^		<0.5	2	4	8	≥16	
A	15	31	25		17	1	1	11	13	
n=43	(34.9)	(72.1)	(58.1)	*mcr-1*			1	9	9	19
				*mcr-2*			1	7	7	15
B	3	20	8		47	1	0	5	3	
n=56	(5.4)	(35.7)	(14.3)	*mcr-1*				2	2	4
				*mcr-2*				3	2	5
C	11	33	44		7	1	1	22	21	
n=52	(21.2)	(63.5)	(84.6)	*mcr-1*			1	15	13	29
				*mcr-2*			1	11	2	14
D	6	27	23		32	0	1	11	11	
n=55	(10.9)	(49.1)	(41.8)	*mcr-1*				7	5	12
				*mcr-2*				2	2	4

a ESBL=Extended-spectrum beta-lactamase,

b SXT=Trimethoprim/sulfamethoxazole and CCL=Colistin. Gray fields represent level of concentration referring resistance

## Discussion

Among the three intensive farms having similar biosecurity management systems (Table-1), farms A and C, where colistin was supplemented in the feed, showed significantly higher rates of colistin resistance (58.1% and 84.6%, respectively) than farm B (14.3%) that did not use colistin (p<0.05). Moreover, in farm B, the gene *mcr-1* was found less frequently than in the other two farms. These results indicated that colistin should be considered as the primary driver for the occurrence of colistin-resistant isolates in conventional swine farms.

Interestingly, although the small natural farm D did not use any antimicrobial agent, *E. coli* isolates collected from this farm showed a high colistin resistance rate of 41.8%. This farm had a distinctively different management regime compared with other farms. Therefore, in addition to antimicrobial usage, there may well be other factors that contribute significantly to AMR bacterial distribution within a farm. Based on the questionnaire survey, the possible source of contamination was found to be animal bedding. The swine pens of farm D were 60 cm deep soil pits filled with rice husk. This alternative bedding material was believed to absorb animal waste and moisture. The rice husk was replaced every 3 months; however, its source was unknown. Contaminated rice husk and soil were the probable biosecurity risks on this farm. Another possible cause for the distribution of AMR bacteria in farm D was its location. This farm was located close to a vegetable cultivating farm where pesticides and fertilizers were commonly used. Animal manure fertilizer could be the source of AMR bacterial contamination. Moreover, bacteria exposed to pesticides can develop resistance to antimicrobial agents faster than those in a typical environment by increasing the efflux pump mechanism or integrating resistant genes to transposable DNA elements [22,23]. However, the sources of resistant isolates could not be elucidated because the AMR isolates were detected in various samples, including feces, floor, boot, and even clean water.

Contamination of AMR bacteria in natural farms has been reported in different food-producing animals in other studies. For example, Rothrock *et al*. [24] reported AMR bacteria in natural broiler farms that did not use antimicrobials in the Southeast United States. Cadena *et al*. [25] detected antimicrobial-resistant genes in soil from plants of organic farms located in Nebraska. Furthermore, Sancheza *et al*. [26] detected antimicrobial-resistant genes from outdoor air samples in organic beef cattle farms. However, several studies comparing the prevalence of AMR bacteria between farm types, such as organic farms, have often reported lower rates than those in conventional farms [27-29].

A standard development of alternative farming is complicated and requires biosecurity measures. An alternative pig production system is trendy for consumers who are concerned about animal welfare and environmental protection. The consumer believes that meat produced through natural farming without the use of antimicrobials is more nutritious and safer. Accordingly, additional research to examine biosecurity risks and subtype of AMR isolates in farm D is mandatory to determine the sources of AMR bacterial contamination.

Most of the colistin-resistant isolates (97%) had a relatively high MIC value (≥8 mg/mL), and 76% of the isolates harbored either *mcr-1* or *mcr-2* gene or both. The detection rates of *mcr-1* and *mcr-2* were not significantly different among the farms (p>0.05). No significant differences were found in the colistin resistance rates during the different production periods in the same farm in this study (p>0.05). However, the number of resistant isolates decreased in every farm when the samples were collected from the finishers, which might be due to the lower use of colistin and other antimicrobial agents in finishers or before slaughtering in conventional swine farms.

Of the 17 tested antimicrobial agents, 15 were “critically important antimicrobials” for human medicine as listed by the World Health Organization [30]. Two agents, cefoxitin and trimethoprim/sulfamethoxazole were grouped as “highly important antimicrobials.” Most of them were high-generation ­antimicrobials, including carbapenems, different generations of cephalosporins, high-generation fluoroquinolones, and aminoglycosides. The majority of these agents have not been commonly used in farm animals but have been used to treat MDR bacterial infection in humans. Previously, the commensal *E. coli* isolates from swine farms in this study were considered to be susceptible to critically important antimicrobial agents. However, the isolates were relatively resistant to colistin, and *mcr-1* and *mcr-2* genes were generally distributed within the farms.

## Conclusion

Commensal *E. coli* isolates in pig farms ­exhibited high colistin resistance rates of 48.5% and a relatively high MIC value of ≥8 mg/mL. Most of the resistant isolates (76%) harbored either *mcr-1* or *mcr-2* gene or both. Colistin use was found to be the primary driver for the increase of colistin-resistant isolates in the three conventional farms. However, in the natural farm free from antimicrobials, colistin-resistant isolates were highly contaminated, indicating the significance of biosecurity risks when developing alternative farm management systems to control AMR bacterial distribution.

## Authors’ Contributions

PK and PA designed the study, conducted the experiment, and wrote the manuscript. PK, ST, and PP performed fieldwork and data collection. CP designed the study and performed statistical analysis. All authors contributed to the drafting and revision of the manuscript. All authors read and approved the final manuscript.
